# Inequities in Filled Overactive Bladder Medication Prescriptions in the US

**DOI:** 10.1001/jamanetworkopen.2023.15074

**Published:** 2023-05-24

**Authors:** Douglas Luchristt, C. Emi Bretschneider, Kimberly Kenton, Melissa Simon, Oluwateniola Brown

**Affiliations:** 1 Department of Obstetrics and Gynecology, Duke University School of Medicine, Durham, North Carolina; 2 Department of Obstetrics and Gynecology, Northwestern University Feinberg School of Medicine, Chicago, Illinois

## Abstract

**Importance:**

Anticholinergic medications to treat overactive bladder (OAB) have been associated with increased risk of cognitive decline, whereas β3-adrenoceptor agonists (hereafter, β3-agonists) have comparable efficacy and do not carry the same risk. Yet, anticholinergics remain the predominant OAB medication prescribed in the US.

**Objective:**

To evaluate whether patient race, ethnicity, and sociodemographic characteristics are associated with receipt of anticholinergic vs β3-agonist OAB medications.

**Design, Setting, and Participants:**

This study is a cross-sectional analysis of the 2019 Medical Expenditure Panel Survey, a representative sample of US households. Participants included individuals with a filled OAB medication prescription. Data analysis was performed from March to August 2022.

**Exposure:**

A prescription for medication to treat OAB.

**Main Outcomes and Measures:**

The primary outcomes were receipt of a β3-agonist or an anticholinergic OAB medication.

**Results:**

An estimated 2 971 449 individuals (mean age, 66.4 years; 95% CI, 64.8-68.2 years) filled prescriptions for OAB medications in 2019; 2 185 214 (73.5%; 95% CI, 62.6%-84.5%) identified as female, 2 326 901 (78.3%; 95% CI, 66.3%-90.3%) self-identified as non-Hispanic White, 260 685 (8.8%; 95% CI, 5.0%-12.5%) identified as non-Hispanic Black, 167 210 (5.6%; 95% CI, 3.1%-8.2%) identified as Hispanic, 158 507 (5.3%; 95% CI, 2.3%-8.4%) identified as non-Hispanic other race, and 58 147 (2.0%; 95% CI, 0.3%-3.6%) identified as non-Hispanic Asian. A total of 2 229 297 individuals (75.0%) filled an anticholinergic prescription, and 590 255 (19.9%) filled a β3-agonist prescription, with 151 897 (5.1%) filling prescriptions for both medication classes. β3-agonists had a median out-of-pocket cost of $45.00 (95% CI, $42.11-$47.89) per prescription compared with $9.78 (95% CI, $9.16-$10.42) for anticholinergics. After controlling for insurance status, individual sociodemographic factors, and medical contraindications, non-Hispanic Black individuals were 54% less likely than non-Hispanic White individuals to fill a prescription for a β3-agonist vs an anticholinergic medication (adjusted odds ratio, 0.46; 95% CI, 0.22-0.98). In interaction analysis, non-Hispanic Black women had an even lower odds of filing a β3-agonist prescription (adjusted odds ratio, 0.10; 95% CI, 0.04-0.27).

**Conclusions and Relevance:**

In this cross-sectional study of a representative sample of US households, non-Hispanic Black individuals were significantly less likely than non-Hispanic White individuals to have filled a β3-agonist prescription compared with an anticholinergic OAB prescription. These differences may reflect an inequity in prescribing behaviors promulgating health care disparities. Targeted research should assess the relative contribution of a variety of individual and societal factors.

## Key Points

**Question:**

Are race, ethnicity, and other sociodemographic characteristics associated with differences in the type of overactive bladder medication used in a nationally representative US survey?

**Findings:**

In this cross-sectional study, after controlling for multiple patient factors, those identifying as non-Hispanic Black were 54% less likely than non-Hispanic White individuals to fill a prescription for a β3-adrenoceptor agonist vs an anticholinergic overactive bladder medication, and these differences were statistically significant.

**Meaning:**

Given the association of anticholinergic overactive bladder medications with the risk of cognitive decline, the observed differences in filled prescriptions may underlie and propagate existing inequities in the US health care system.

## Teaser

This cross-sectional study evaluates whether patient race, ethnicity, and sociodemographic characteristics are associated with receipt of anticholinergic vs β3-agonist medications to treat overactive bladder.

## Introduction

Overactive bladder (OAB) is a highly prevalent chronic condition that substantially affects the quality of life of individuals with it. In survey-based epidemiologic studies,^1^ up to 27% of men and 43% of women reported bothersome OAB symptoms. Medication therapy is a mainstay in the treatment algorithm for OAB and is recommended as second-line therapy for those for whom behavioral interventions fail.^2^ There are 2 classes of oral medications currently used in OAB treatment: antimuscarinic (anticholinergic) and β3-adrenoceptor agonists (hereafter, β3-agonists). In addition to having higher rates of bothersome adverse effects, including dry eyes, dry mouth, and constipation, there has been an expanding body of literature associating anticholinergic use with negative long-term cognitive effects, including dementia.^3,4,5,6,7,8,9^

Although there is no established causal relationship between anticholinergics and dementia, the association data are compelling. A recent systematic review and meta-analysis^8^ found a 1.46 risk ratio for a new diagnosis of dementia with anticholinergic exposures, comparable to as little as 3 months of use of oxybutynin, a common anticholinergic OAB medication. In contrast, β3-agonists have been shown to have a more favorable tolerability profile compared with anticholinergics, including lowered risk of cognitive adverse effects.^10,11^ Because of the potential short-term and long-term cognitive effects, the American Urologic Association advises caution in the use of anticholinergic medications to treat elderly and frail individuals with OAB,^2^ and the American Geriatrics Society Beers Criteria has listed anticholinergic OAB medications as a potentially inappropriate medication for elderly individuals, considering disease and syndrome interactions.^12^ Meanwhile, in 2020, the American Urogynecologic Society (AUGS) updated its clinical consensus statement and explicitly recommended avoiding anticholinergic medications to treat OAB in individuals older than 70 years,^13^ although, notably, several studies have demonstrated increased dementia risk with exposure below this age threshold.^8^ Despite the concerning association of anticholinergics with dementia, prescribing patterns still overwhelmingly favor anticholinergic medications.^14^

Studies identifying patient populations that may be at risk for inequities related to medical therapies for OAB and the structural factors underlying those inequities are lacking.^15^ We aimed to evaluate whether patient racial and ethnic and sociodemographic identities are associated with receipt of anticholinergic and β3-agonist bladder medications using a nationally representative survey of US households. We hypothesized that significant differences would be seen according to race and ethnicity and that non-Hispanic White individuals would have greater odds of receiving β3-agonist OAB medications compared with other groups. Our choice to evaluate racial differences is deliberate. Race is a social construct that is a proxy for many underlying causal factors, including health care access and societal norms and laws that influence the resource allocation that impacts health outcomes.^16,17^ Because certain populations in the US have higher rates of dementia, especially non-Hispanic Black individuals compared with their White counterparts and women compared with men,^18,19,20^ it is important to elucidate whether these groups are structurally at risk for medication use that is associated with dementia.

## Methods

We performed a cross-sectional analysis using the 2019 Agency for Healthcare Research and Quality (AHRQ) Medical Expenditure Panel Survey (MEPS).^21^ The study was reviewed by the Northwestern University institutional review board and was deemed to be not human participants research because the data were anonymous and publicly available; thus, informed consent was not required for this specific analysis, in accordance with 45 CFR §46. This study follows the Strengthening the Reporting of Observational Studies in Epidemiology (STROBE) reporting guidelines for cross-sectional studies.

MEPS is administered by the AHRQ. Data are collected from a representative sample of US households, documenting utilization and associated costs of health care in the US. Notably, along with the completion of the MEPS survey tools, AHRQ also gathers participants’ permission to query health care institutions to verify data. Pharmacies’ records are extracted to ascertain the type, dosage, and payments associated with each filled prescription during the survey period.^21^ These supplemental data are cross-sectional in nature and do not capture prescriptions outside of the 2019 survey period. Each prescription is assigned a therapeutic class according to the Multum Lexicon.^22^

All prescriptions for an OAB medication (drug therapeutic class 264, urinary antispasmodic) were identified from the 2019 Prescribed Medicines File. The primary outcome was an individual’s receipt of an antimuscarinic (herein referred to as anticholinergics) or β3-agonist OAB medication. We also collected the name of the specific medication filled, along with the quantity, out-of-pocket cost, and reported date that the medication was first started. Receipt of each medication class was not mutually exclusive, because individuals may have switched between medication classes within the survey period or may have received combination therapy with both.

Prescription records were linked to the individual record within the MEPS Full-Year Consolidated Data file,^23^ which contains individual and household survey data. On the basis of prior literature describing disparities in OAB treatment, as well as the relative contraindications for each class of medication, we a priori selected a series of variables of interest that would likely be associated with the receipt and type of OAB medications. These included race and ethnicity, poverty level, insurance status, educational attainment, and sex; given potential medical contraindications to either anticholinergic or β3-agonist medications, we also adjusted for diagnosis of high blood pressure and cognitive impairment.^6,8,9,10,11,24,25^ All variables were prespecified in the MEPS consolidated data file. Specifically, race and ethnicity were self-selected within the MEPS survey instrument, and we report MEPS-derived racial categories within our analysis. Racial and ethnic categories were defined by MEPS (ie, Hispanic, non-Hispanic Asian, non-Hispanic Black, non-Hispanic White, and multiple or other races; please note that the MEPS data dictionary does not specify which racial identities were collapsed into the other category); no modification or combination of the racial categories was made for this analysis.^21^ Hypertension and cognitive impairment were self-reported by individuals or their family members within the household survey (variables DFCOG42 and HIBPDX).

MEPS uses skip logic and branching, yielding results of *not applicable* for some measures, which were grouped with *no* in this analysis. Only those entries explicitly coded as missing were retained as such.

### Statistical Analysis

Data analysis was performed from March to August 2022. National weighted count estimates were generated using the provided survey weights and variance estimates within the Full-Year Consolidated Data file (variables VARSTR, VARPSU, and PERWT18F). The descriptive analysis assessed the characteristics of the population who had at least 1 filled prescription for an OAB medication, as well as those specifically filling an anticholinergic or β3-agonist medication prescription or both. Multinomial regression was used to estimate the odds of receipt of an anticholinergic or β3-agonist monotherapy. In addition, interaction terms were used to assess for sex-specific differences, because patient sex is a factor known to be associated with increased risk of differential medication use.^14^ Point estimates and 95% CIs were generated for reported odds ratios (ORs). No other formal statistical testing was performed. SAS statistical software version 9.4 (SAS Institute) was used for all analyses, using the PROC Surveymeans, PROC Surveyfreq, and PROC Surveylogistic commands. Two sets of sensitivity analyses were performed. A sensitivity analysis was performed using 70 years (as opposed to 65 years) as the dichotomous cutoff for senior age status to align with AUGS clinical guidelines. In addition, given the potential association of anticholinergic medication use with cognitive decline, we also performed a sensitivity analysis excluding cognitive decline from the multivariable model because of a possible collider effect.

## Results

Nationally, an estimated 2 971 449 individuals (mean age, 66.4 years; 95% CI, 64.8-68.2 years) filled prescriptions for urinary antispasmodic medications in 2019, with 2 229 297 individuals (75.0%) filling an anticholinergic medication prescription, 590 255 (19.9%) filling a β3-agonist prescription, and 151 897 (5.1%) filling prescriptions for both medication classes. Population characteristics are presented in Table 1. The majority of individuals filing an OAB medication identified as women (2 185 214 individuals; 73.5%; 95% CI, 62.6%-84.5%). With respect to race and ethnicity, the majority identified as non-Hispanic White (2 326 901 individuals; 78.3%; 95% CI, 66.3%-90.3%), with the next largest groups identifying as non-Hispanic Black (260 685 individuals; 8.8%; 95% CI, 5.0%-12.5%), Hispanic (167 210 individuals; 5.6%; 95% CI, 3.1%-8.2%), multiple races or other race (158 507 individuals; 5.3%; 95% CI, 2.3%-8.4%), and non-Hispanic Asian (58 147 individuals; 2.0%; 95% CI, 0.3%-3.6%). All identified records were included within the analysis, although notably only 1 surveyed individual filling an OAB medication was uninsured, and that individual filled an anticholinergic prescription only. The most commonly filled medication was oxybutynin, followed by mirabegron and tolterodine (Table 2). Out-of-pocket costs were substantially different between medication classes, with β3-agonists having a median out-of-pocket cost of $45.00 (95% CI, $42.11-$47.89) per prescription fill compared with a median of $9.78 (95% CI, $9.16-$10.42) for anticholinergics.

**Table 1.** Characteristics of Individuals Filling an OAB Medication Prescription by Medication

Characteristic	Individuals, No. (%) [95% CI]			
	Any OAB medication	Anticholinergic monotherapy	β3-agonist monotherapy	Dual anticholinergic and β3-agonist therapy
Individuals, estimated total No. (95% CI)	2 971 449 (2 553 351-3 389 546)	2 229 297 (1 943 233-2 515 360)	590 255 (444 023-736 486)	151 897 (81 668-222 125)
Cognitive decline^a^				
Yes	707 750 (23.8) [16.1-31.6]	555 576 (24.9) [15.9-34.0]	102 301 (17.3) [5.5-29.2]	49 873 (32.8) [5.0-60.7]
No	2 263 699 (76.2) [64.3-88.0]	1 673 720 (75.1) [60.8-89.3]	487 954 (82.7) [56.6-100.0]	102 024 (67.2) [26.7-107.6]
Educational attainment				
Less than a high school diploma	567 830 (19.1) [13.5-24.7]	426 552 (19.1) [12.7-25.6]	117 016 (19.8) [6.0-33.6]	2426 (1.6) [0.0-21.3]
High school diploma or general educational development	913 743 (30.8) [23.6-37.9]	711 773 (31.9) [23.6-40.2]	164 149 (27.8) [12.7-42.9]	37 821 (24.9) [2.4-47.4]
More than a high school diploma	1 489 876 (50.1) [40.1-60.2]	1 090 972 (48.9) [36.7-61.1]	309 090 (52.4) [31.4-73.3]	89 813 (59.1) [21.7-96.5]
High blood pressure				
Yes	2 095 280 (70.5) [59.0-82.0]	155 0371 (69.5) [56.4-82.7]	410 314 (69.5) [45.3-93.7]	134 594 (88.6) [40.9-136.3]
No	876 169 (29.5) [22.1-36.8]	678 925 (30.5) [21.3-39.6]	179 941 (30.5) [14.2-46.8]	17 303 (11.4) [0.0-24.9]
Insurance coverage				
Private	1 615 235 (54.4) [44.2-64.5]	1 181 228 (53.0) [40.2-65.8]	366 401 (62.1) [40.9-83.3]	67 605 (44.5) [11.3-77.7]
Public	1 349 768 (45.4) [36.4-54.4]	1 041 622 (46.7) [36.0-57.4]	223 854 (37.9) [21.5-54.4]	84 292 (55.5) [20.7-90.3]
Uninsured	6446 (0.2) [0.0-0.6]	6446 (0.3) [0.0-0.9]	No observations	No observations
Poverty category^b^				
Poor	445 575 (15.0) [10.2-19.8]	349 809 (15.7) [9.4-22.0]	70 691 (12.0) [3.7-20.2]	25 075 (16.5) [0.0-33.1]
Near poor	160 964 (5.4) [3.0-7.8]	116 348 (5.2) [2.4-8.0]	38 774 (6.6) [1.0-12.1]	5842 (3.8) [0.0-11.4]
Low income	346 755 (11.7) [8.0-15.4]	270 226 (12.1) [7.4-16.9]	61 922 (10.5) [1.9-19.1]	14 607 (9.6) [0.0-28.5]
Middle income	871 085 (29.3) [21.9-36.8]	687 874 (30.9) [21.8-39.9]	147 760 (25.0) [9.6-40.5]	35 450 (23.3) [2.7-44.0]
High income	1 147 071 (38.6) [30.0-47.2]	805 039 (36.1) [26.1-46.1]	271 108 (45.9) [26.2-65.6]	70 923 (46.7) [12.1-81.3]
Race and ethnicity				
Hispanic	167 210 (5.6) [3.1-8.2]	123 853 (5.6) [2.6-8.6]	27 746 (4.7) [0.6-8.8]	15 611 (10.3) [0.0-25.3]
Non-Hispanic Asian	58 147 (2.0) [0.3-3.6]	36 837 (1.7) [0.0-3.4]	21 309 (3.6) [0.0-8.8]	No observations
Non-Hispanic Black	260 685 (8.8) [5.0-12.5]	229 776 (10.3) [5.5-15.1]	27 450 (4.7) [0.5-8.8]	3459 (2.3) [0.0-6.7]
Non-Hispanic other race or multiple races^c^	158 507 (5.3) [2.3-8.4]	117 274 (5.3) [1.8-8.7]	41 232 (7.0) [0.0-15.7]	No observations
Non-Hispanic White	2 326 901 (78.3) [66.3-90.3]	1 721 557 (77.2) [62.9-91.5]	472 518 (80.1) [54.9-100.0]	132 827 (87.4) [39.1-100.0]
Senior age status, y				
<65	863 547 (29.1) [21.5-36.6]	694 973 (31.2) [21.7-40.6]	137 128 (23.2) [10.7-35.8]	31 446 (20.7) [0.0-45.2]
≥65	2 074 046 (69.8) [58.3-81.3]	1 519 121 (68.1) [55.5-80.8]	434 474 (73.6) [48.0-99.2]	120 451 (79.3) [35.0-100.0]
Sex^d^				
Male	786 235 (26.5) [19.4-33.6]	573 203 (25.7) [17.7-33.8]	171 274 (29.0) [9.8-48.3]	41 758 (27.5) [1.6-53.3]
Female	2 185 214 (73.5) [62.6-84.5]	1 656 094 (74.3) [61.1-87.5]	418 981 (71.0) [49.0-92.9]	110 139 (72.5) [29.0-100.0]

Abbreviations: MEPS, Medical Expenditure Panel Survey; OAB, overactive bladder.

^a^ Cognitive decline was self-defined by the individual or family member within the survey instrument (variable DFCOG42).

^b^ Defined by the MEPS survey.^23^

^c^ Other (the MEPS data dictionary does not specify which racial identities were collapsed into the other category) combination was performed by the MEPS survey and was included in variable RACETHX.^23^

^d^ Sex is defined within the MEPS survey in variable SEX; nonbinary classifications are not provided.

**Table 2.** Types of OAB Medications Defined by the Lexi-Comp Database

Medication type	Filled OAB medication prescriptions, No. (%) [95% CI]
Oxybutynin	1 476 858 (49.7) [39.4-60.0]
Mirabegron	742 152 (25) [19.6-30.4]
Tolterodine	387 878 (13.1) [8.3-17.8]
Solifenacin	317 209 (10.7) [6.8-14.6]
Other anticholinergic^a^	332 350 (11.2) [7.0-15.3]

Abbreviation: OAB, overactive bladder.

^a^ Data are shown as reported in the data set. No modification or combination of medication types were performed by the study authors.

In multivariable analyses incorporating those factors determined a priori to be potential contributors to differential oral pharmacologic OAB treatment, race was the only factor significantly associated with receipt of each medication class. For example, non-Hispanic Black individuals were 54% less likely than non-Hispanic White individuals to fill a β3-agonist as opposed to an anticholinergic medication (adjusted OR, 0.46; 95% CI, 0.22-0.98) (Table 3). Moreover, there was a significant interaction noted between race and sex, with non-Hispanic Black women having 90% lower odds of filling a β3-agonist prescription as opposed to an anticholinergic medication as monotherapy (adjusted OR, 0.10; 95% CI, 0.04-0.27) compared with non-Hispanic White women. Among men, the differences followed similar trends for those of non-Hispanic Black racial and ethnic categorization, although the 95% CIs crossed unity. No significant covariates were identified among those receiving dual therapy, compared with anticholinergic therapy alone, although the small portion of the sample receiving dual therapy limits our ability to generate precise estimates. Sensitivity analyses (data not reported) evaluating a dichotomous age cutoff of 70 years (as opposed to 65 years) and excluding cognitive impairment as a covariate showed no meaningful difference in findings.

**Table 3.** Adjusted ORs for Race and Ethnicity and Filling a β3-Agonist Monotherapy vs Anticholinergic Monotherapy Prescription for Overactive Bladder Medication Among All Individuals, by Sex

Race and ethnicity categorization	Adjusted OR (95% CI)^a^		
	All individuals	Men	Women
Hispanic	1.08 (0.34-3.46)	0.24 (0.02-2.79)	1.52 (0.30-7.69)
Non-Hispanic Asian	2.49 (0.48-12.84)	3.58 (0.23-56.57)	1.42 (0.14-14.31)
Non-Hispanic Black	0.46 (0.22-0.98)	0.73 (0.21-2.54)	0.10 (0.04-0.27)
Non-Hispanic other race or multiple races^b^	1.67 (0.43-6.50)	0.54 (0.11-2.58)	1.54 (0.27-8.83)
Non-Hispanic White	1 [Reference]	1 [Reference]	1 [Reference]

Abbreviation: OR, odds ratio.

^a^ Adjusted ORs were derived from multivariable logistic regression model controlling for race and ethnicity, poverty level, insurance status, educational attainment, sex, diagnosis of high blood pressure, and cognitive impairment.

^b^ Other race (the Medical Expenditure Panel Survey data dictionary does not specify which racial identities were collapsed into the other category) and multiple races were calculated by the Agency for Healthcare Research and Quality and reported in variable RACETHX.^23^

## Discussion

By use of the MEPS, a nationally representative sample of US households, this cross-sectional analysis found that individuals who self-identified as non-Hispanic Black were significantly less likely to fill a prescription for a β3-agonist prescription vs anticholinergic OAB medication compared with non-Hispanic White respondents, after controlling for income, insurance coverage, educational attainment, and potential medical contraindications to either medication class. These differences were most pronounced among those who identified as Black women, with a 90% lower odds of a β3-agonist use compared with an anticholinergic OAB medication as monotherapy. Although prior research^25^ has demonstrated disparities in receipt of third-line OAB therapy (intravesical onabotulinum toxin injections, percutaneous nerve stimulation, and sacral neuromodulation) on the basis of patient race and ethnicity, to our knowledge, no studies have assessed for racial and ethnic differences in prescriptions for OAB medications.

Our findings are akin to documented differences in access to prescription drugs for other conditions described in the literature^26,27,28,29^ and are suggestive of a lack of pharmacoequity in the medical management of OAB. The term *pharmacoequity*, coined by Essien et al,^29^ refers to the goal of “ensuring that all individuals, regardless of race and ethnicity, socioeconomic status or availability of resources, have access to the highest quality medications to manage their health.” The observed differences in our analysis of higher odds of anticholinergic use compared with β3-agonist use represent an inequity not because of medication efficacy but given the risks associated with anticholinergic medications.

Accumulating evidence suggests a consistent association between anticholinergic medication use and increased risk of cognitive decline and dementia,^3,4,5,6,7,8,9,11,13^ whereas β3-agonists do not demonstrate the same adverse effect profile.^10,11^ In contrast, anticholinergic medication exposure further appears to increase the risk of progression of mild cognitive impairment and Alzheimer disease.^30^ Meanwhile, initiation of β3-agonist medication, compared with anticholinergic medication, for the treatment of OAB is associated with a reduced risk of subsequent dementia. Welk et al^11^ evaluated the risk of dementia in 47 324 new users of anticholinergic medications compared with 23 662 new users of a β3-receptor agonist for the treatment of OAB. They found an increased risk of dementia among anticholinergic users compared with β3-agonist users (hazard ratio, 1.23; 95% CI, 1.12-1.35).^11^

Multiple studies^31,32^ also report that the risk of dementia is higher in non-Hispanic Black individuals than in non-Hispanic White individuals in the US. Furthermore, a recent study^20^ found no evidence of a decrease in the racial disparities in dementia prevalence or incidence in adults aged 70 years or older in the US from 2006 to 2016. Because there are no disease-modifying treatments for the most common forms of dementia, the declining overall prevalence and incidence of dementia observed in population studies are thought to be attributed to changing risk factor exposure.^20^ Medication exposure that can add to the risk of dementia is a possible modifiable risk factor. Given comparable efficacy among anticholinergic and β3-agonist medications for both objective and subjective disease measures,^33^ initial treatment of OAB with a β3-agonist medication is prudent. In fact, a 2019 cost-benefit analysis suggested that there were overall lower health care utilization and costs when a β3-agonist prescription was provided compared with an anticholinergic OAB medication.^34^

The factors associated with these disparities remain unclear, because numerous barriers to accessing β3-agonist medications exist and likely contribute to the observed differences in our studies, one of which is insurance coverage. It is estimated that 29 million US residents, many of whom identify as Black and Hispanic, do not have insurance and lack prescription coverage.^35^ Frequently, insurance providers and prescription drug plans require prior authorization and/or failure of 1 or more anticholinergic medications before initiation of a β3-agonist. This includes the Veterans Affairs^36^ and TRICARE^37^ health systems, as well as many of the country’s largest private insurance groups.^38,39^ Even when a β3-agonist is included in the insurance formulary, patients typically face higher copayments and deductibles, leading to potential financial barriers, as demonstrated by the greater than 2-fold higher median out-of-pocket cost associated with β3-agonists observed within this study. We attempted to control for these potential financial and institutional barriers by controlling for both income level and insurance status within our multivariable models, with observed racial disparities persisting. This aligns with results from a 2020 analysis of a US commercial insurance claims database showing that any race other than White was significantly associated with decreased odds of third-line treatment for OAB (adjusted OR, 0.89; 95% CI, 0.87-0.91).^25^

Other factors potentially contributing to the observed differences in this study may be patient symptoms, experience of adverse effects, and stability on medication therapy, all of which may mediate the frequency of medication reevaluation. For example, if an individual is started on an anticholinergic and experiences adequate symptom relief, the individual may not seek care for medication revision because they may be hesitant to try another medication. However, individuals identifying as Black in the US have been shown to have rates of bothersome OAB symptoms comparable to or higher than those for White individuals,^40,41^ and their care-seeking behaviors are also comparable.^15^ Furthermore, our analysis only included individuals who filled a prescription medication for OAB. Although non-Hispanic Black individuals were underrepresented in many of the pivotal studies of both anticholinergic and β3-agonist OAB medications,^2,42,43^ there is no evidence of differential efficacy or adverse event incidence or tolerability based on racial identity. In our analysis, we could not assess and control for disease severity or prior treatment using the MEPS database; however, multivariable analyses did control for other factors known to be associated with disease severity and treatment, including age, education, insurance status, and income, as well as medical comorbidities such as hypertension and preexisting cognitive impairment, which may provide relative contraindications to one class of medication.^25,44^

Finally, race, which is a social and political construct, has high concordance with other structural determinants of health, including economic deprivation, education, wealth, and geography.^45,46^ Thus, it is critical to acknowledge the role that structural racism plays as a root cause of racial health inequities.^47,48,49,50,51,52^ The resultant impact on health materializes through multiple pathways, including but not limited to the restriction of education opportunities, economic inequities (which, downstream, determine things like adequate insurance coverage),^53^ and limited access to health care facilities including pharmacies. Prescription drug prices vary substantially by geography and across the type of retail pharmacy, with significantly higher out-of-pocket prices at independent pharmacies.^54^ Limited pharmacy access in neighborhoods may expose residents from marginalized communities to higher drug prices at independent pharmacies, which can exacerbate inequities.^29^ Given the limitations of the MEPS data, geographic information was not available to be assessed within this analysis.

### Limitations

We wish to acknowledge and address several important limitations of our analysis. Given the nature of the MEPS data collection, we are only able to describe patterns in filled OAB medications and cannot assess motivations for the types of prescriptions prescribed to and filled by patients, which may be associated with practitioner preference, national medication availability, insurance formularies, and expected out-of-pocket costs (all of which are not ascertainable within these data), as well as medication adherence. Nonetheless, these data describe differences in receipt of OAB medications, which, in turn, can have disparate outcomes irrespective of the proximal cause. We are also limited by the observational nature of the data, as well as the methods of the MEPS sampling. Although we have used recommended analytic techniques for the assessment of these complex survey data, the observed differences are limited to associations and may be influenced by unobserved biases affecting survey participation or administration. The MEPS database does not provide information on prescriber specialty, although notably, prior analyses have suggested that primary care practitioners are the largest prescribers of OAB medications.^55^ Further dedicated, prospective research is indicated to assess the myriad factors influencing both practitioner and patient decisions surrounding prescription and medication fills. In addition, we have used the most recent set of survey data from 2019, which notably was collected before changes in the AUGS guidelines and Food and Drug Administration approval of a second β3-agonist for OAB.^56^ Current practice patterns may have been influenced by these events and cannot be ascertained by our data.

## Conclusions

In this cross-sectional study of a representative sample of US households, when controlling for multiple patient factors, non-Hispanic Black individuals in the US were found to be significantly less likely to have filled a prescription for a β3-agonist and significantly more likely to have filled a prescription for an anticholinergic OAB medication compared with non-Hispanic White individuals. This disparity was most pronounced among women. Given the high prevalence of this chronic condition and growing evidence linking prolonged anticholinergic medication exposure with cognitive decline and dementia, these findings highlight important potential implications of current OAB medication treatment patterns. Furthermore, the observed disparities in these prescription fill patterns may underlie existing health inequities within the US health care system with respect to OAB outcomes. Targeted research is needed to assess the relative contribution of a variety of individual-level and societal-level factors contributing to these observed disparities.

## Notes

Supplement.
Data Sharing Statement
