# Supplementary material for: Inequities in Filled Overactive Bladder Medication Prescriptions in the US
Source: JAMA Netw Open. 2023 May 24;6(5):e2315074. doi: 10.1001/jamanetworkopen.2023.15074 (PMC10209743; doi:10.1001/jamanetworkopen.2023.15074)
Supplement: Supplement. — Data Sharing Statement [file jamanetwopen-e2315074-s001.pdf]

Data Sharing Statement

Luchristt. Inequities in Filled Overactive Bladder Medication Prescriptions in the US. *JAMA Netw Open*. Published May 24, 2023. doi:10.1001/jamanetworkopen.2023.15074

Data
Data available: Yes
Data types: Deidentified participant data, Data (not involving human participants)
How to access data: MEPS data utilized for this analysis are publicly available at www.meps.ahrq.gov.
When available: With publication

Supporting Documents
Document types: Statistical/analytic code
How to access documents: Upon request to douglas.h.luchristt@gmail.com
When available: With publication

Additional Information
Who can access the data: Researchers whose proposed use of the data has been approved
Types of analyses: Research purposes
Mechanisms of data availability: After author's approval of a proposal
